# Association between neurodegenerative plasma biomarkers and geriatric depression in older adults with and without clinical dementia in Kinshasa, Democratic Republic of the Congo

**DOI:** 10.3389/fpsyt.2026.1787247

**Published:** 2026-05-22

**Authors:** Nathan Tsengele, Alfred Sodi Magudigana, Guy Gikelekele, Emmanuel Epenge, Guy Kangula, Eric Muteba Vukumuna, Abraham Mifundu Bilongo, Said Mbuku Nguala, Degani Banzulu Bomba, Gilbert Mananga Lelo, Adelin N’situ Mankubu, Jean Ikanga

**Affiliations:** 1 Department of Psychiatry, Faculty of Medicine, University of Kinshasa, Kinshasa, Democratic Republic of Congo; 2 Faculty of Medicine, Kikwit University, Kikwit, Democratic Republic of Congo; 3 Kinshasa Memory Clinic, Kinshasa, Democratic Republic of Congo; 4 Faculty of Medicine, University of Bandundu, Bandudu, Democratic Republic of Congo; 5 Department of Neurology, University of Kinshasa, Kinshasa, Democratic Republic of Congo; 6 Faculty of Medicine, Protestant University of Congo, Kinshasa, Democratic Republic of Congo; 7 Department of Rehabilitation Medicine, Emory University School of Medicine, Atlanta, GA, United States

**Keywords:** plasma biomarkers, GFAP, NfL, dementia, geriatric depression, Kinshasa, sub-Saharan Africa

## Abstract

**Introduction:**

Previous Western studies have shown an association between neurodegenerative plasma biomarkers and geriatric depression. However, few studies have examined this relationship in sub-Saharan African countries. This article examines this association in older adults with or without clinical dementia in Kinshasa, Democratic Republic of Congo.

**Methods:**

This cross-sectional study included eighty-one people aged 65 years and older assessed for dementia using the Community Dementia Screening Instrument (CSID) and the Alzheimer’s Disease Questionnaire. Depression and cognitive impairment were assessed using the Geriatric Depression Scale and the African Neuropsychological Battery (ANB), respectively. Plasma biomarkers measured included β-amyloid (Aβ) 40/42, phosphorylated tau 181 (p-tau), neurofilament light chain (NfL), glial fibrillar acid protein (GFAP), interleukins (IL-1β, IL-10), and tumor necrosis factor-α (TNF-α). Logistic regression models were used to estimate the likelihood that these biomarkers are associated with depression, adjusting for sex, age, and education level.

**Results:**

The mean age of the participants was 72.9 years. Of those suspected of suffering from Alzheimer’s disease (AD), 80% had geriatric depression. High blood pressure was common in participants with suspected AD (60%). People with dementia had significantly lower cognitive scores on all ANB tests than those without dementia. GFAP and NfL were significantly associated with higher odds of geriatric depression, with odds ratios of 1.98 (1.17–3.67; p = 0.02) and 1.76 (1.06–3.20; p = 0.04), respectively. Other biomarkers were not associated with depression.

**Conclusion:**

This study suggests a potential role of nonspecific biomarkers of AD, neurodegeneration, and astrocytic glial activation in geriatric depression in older adults in sub-Saharan Africa.

## Introduction

Depressive symptoms occur in up to 90% of older adults with dementia, particularly in Alzheimer’s disease (AD) (1–4), a condition characterized primarily by cognitive deficits and behavioral impairment associated with specific neuropathological biomarkers (4–6). Similar symptoms are also common in late-onset depression, where impairments in memory, attention, and executive functions frequently appear. Conversely, people with AD may experience emotional and behavioral disturbances similar to those seen in geriatric depression. These symptoms contribute significantly to morbidity in both patients and caregivers (7, 8). Although several studies suggest that depression is more prevalent in dementia with Lewy bodies and Parkinson’s disease than in AD (9), the overlap between depressive symptoms and neurodegenerative processes remains an important area of investigation.

Research has identified several neuropathological mechanisms that may explain this association (9). Of these, excessive production and brain accumulation of AD-related neurodegenerative biomarkers have been implicated (10–14). Although relatively rare, most Western studies reporting significant associations between biomarkers and depressive symptoms have focused on AD-specific markers such as amyloid proteins (11–17) and tau proteins (13, 18–20). However, other studies have also highlighted associations with inflammatory and nonspecific biomarkers, including neurofilament light chain (NfL) (21–23), glial fibrillar acid protein (GFAP) (23, 24), and inflammatory cytokines such as IL-6 and TNF-α (25, 26).

A systematic review and meta-analysis conducted by Nascimento et al. (2015) showed that elderly subjects with late-onset depression had a higher plasma Aβ40/Aβ42 ratio than non-depressed participants, as well as a marginally significant reduction in Aβ42 concentrations in the cerebrospinal fluid (27). In addition, Pamora et al. (2022) demonstrated that a reduced plasma Aβ42/Aβ40 ratio is consistently associated with the diagnosis of depression, and that increased severity of depression at baseline predicts a decrease in this ratio at three years (28). In addition, an earlier study by Pamora et al. (2012) found significantly lower levels of amyloid peptide-β42 in subjects with major depression compared to controls, suggesting a possible increase in brain amyloid deposits (17). In addition, high levels of Aβ have been shown to be associated with a 4.5-fold increase in the likelihood of clinically significant depressive symptoms (11). A positive correlation was also observed between the severity of depression and amyloid plaque load (16). A study conducted by Babulal et al. (2020) reported that high levels of tau protein double the risk of depression, suggesting a stronger relationship between tau pathology and depressive symptoms than between amyloid and depression (13). In addition, sadness and anxiety have been associated with tau accumulation in women, and higher tau concentrations in the lower temporal region are correlated with higher scores on the Geriatric Depression Scale (19, 20).

Other research has shown that NfL is associated with depression. For example, Bavato et al. (2021), found significantly higher levels of NfL in depressed individuals compared to healthy controls, suggesting widespread axonal injury or loss indicative of active neurodegeneration (21). Another study reported that depression or anxiety is associated with elevated plasma NfL levels in people with mild cognitive impairment (MCI) and AD (29). GFAP levels also increase with age and severity of depression, supporting the role of neuroinflammation in depressive pathophysiology (24, 30). GFAP has also been identified as a marker of future neuropsychiatric symptoms and their severity (23). Serum GFAP may improve the differential diagnosis of major depressive disorder (MDD), objectively quantify the severity of depression and help monitor astroglial pathology in MDD (24). In addition, central and peripheral inflammation may contribute to the neuropsychiatric symptoms of neurodegenerative dementias, with elevated levels of IL6 and TNFα associated with depressive symptoms (25).

Although many Western studies have linked neurodegeneration to depression in older adults, including using positron emission tomography and CSF biomarkers, these results are almost non-existent for populations in sub-Saharan Africa (SSA). The originality of this study lies in its anchoring in a sub-Saharan African population, which is still largely underrepresented in the scientific literature. This approach allows to explore the interactions between depression and AD biomarkers in an environmental, socio-cultural and health context distinct from those of Western countries, thus contributing to filling an important data gap in this region.

Given the high prevalence of depression previously observed in our Kinshasa cohort (31), this study aims to evaluate the association between plasma neurodegenerative biomarkers and depression in older Congolese adults.

We hypothesize that specific plasma biomarkers (Aβ, p-tau) are significantly associated with depression in this population, regardless of clinical dementia status, thus offering potentially accessible diagnostic tools for low-resource settings.

## 2 Methods

### 2.1 Study area

This study was conducted between October 2019 and December 2022 in Kinshasa, a large cosmopolitan and culturally diverse city. Kinshasa was chosen as the study setting because of its demographic variability (32–34). Other methodological details have been published in our previous work (31, 35).

### 2.2 Population

This cross-sectional study included participants aged 65 years or older, selected from our preliminary dementia prevalence study (26). Based on inclusion criteria such as: (1) Be 65 years of age or older (to increase the likelihood of including people with dementia) and reside in the study area; (2) Speak French or one of the four national languages; (3) have a caregiver serving as an informant; (4) No significant hearing and visual deficit that could hinder the execution of the task; (5) Capacity of the subject or family to give informed consent. Subjects who did not meet the inclusion criteria were excluded as described in our previous publications (31, 36) and 1432 eligible participants were identified.

Due to the lack of validated diagnostic criteria for dementia in sub-Saharan Africa, we used the Alzheimer’s Questionnaire (AQ) (37) and the Community Dementia Screening Instrument (CSID) (38) to determine the neurological status of participants. AQ assesses daily functioning and symptoms related to Alzheimer’s disease, while CSID — widely used in international and African studies, including in Central Africa, Nigeria, and South Africa — assesses cognitive abilities (39–42).

In addition to the cognitive and functional deficits defined by the Diagnostic and Statistical Manual of Mental Disorders, Fifth Edition, Text Revision (DSM-5TR) (43), we applied validated CSID cut-offs in Brazzaville, a city geographically and culturally close to Kinshasa (42). As in our previous study (36), participants were classified into four groups based on CSID and AQ scores:

- Major Neurocognitive Disorder (Dementia)
- Mild Neurocognitive Impairment (MCI)
- Subjective cognitive impairment
- Healthy cognitively normal (HC) controls

A total of 1,161 participants classified as having MCI, or subjective cognitive impairment, were excluded. For AQ, only the total score was used; A score ≥13 out of 27 indicated probable dementia (44). In addition, alcohol and tobacco use corresponded to recent or previous use, as reported by participants. These data were collected in a declarative manner and were not objectively quantified.

### 2.3 Procedure

Participants underwent a comprehensive clinical assessment including cognitive tests, self-report questionnaires, and standard psychiatric and neurological assessments. A diagnostic panel consisting of a neurologist (EE), a psychiatrist (GG) and a neuropsychologist (JI) established the presence of dementia and depression.

Participants then completed the Geriatric Depression Scale (GDS) and cognitive assessments, including selected subtests from the African Neuropsychological Battery (ANB) (45). Blood samples were collected by a phlebotomist trained at the Kinshasa Medical Center (CMK).

A diagnosis of dementia was confirmed in 56 individuals, who were matched with 58 cognitively healthy controls according to age, sex and education level. The participants were finally divided into two groups:

- Healthy controls (HC): no cognitive impairment on CSID or ANB and normal functioning on AQ.
- Dementia: impairment in at least two cognitive domains (scores ≥1.5 SDs below average) and functional impairment according to AQ criteria.

Particular attention was paid to the identification of profiles compatible with Alzheimer’s disease, characterized by a low learning slope, a marked memory impairment and multiple severe cognitive deficits during CSID or ANB tests, as well as functional decline on AQ.

Medical history was self-reported using “yes,” “no,” or “don’t know” answers to standardized questions administered by clinical psychologists or trained medical interns. All participants provided written informed consent and were compensated for their time.

**Figure 1:**
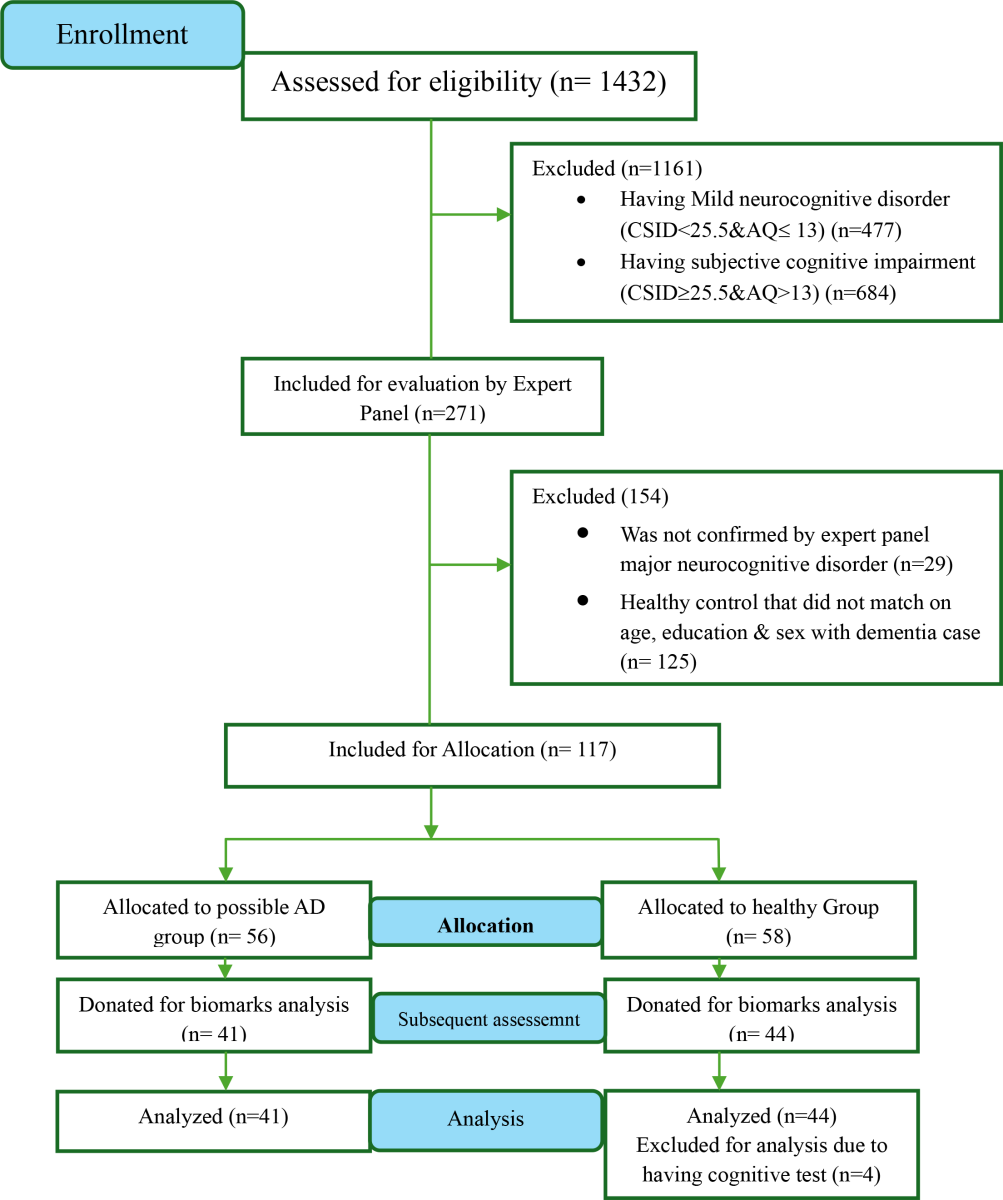
Flow diagram of participant classifications using the CSID and the AQ in the current study. CSID, community screening interview for dementia; AQ, Alzheimer’s Questionnaire; AD, Alzheimer’s desease.

Plasma biomarkers were collected from 44 participants with dementia and 41 healthy controls. Four individuals were excluded due to missing cognitive data, resulting in a final sample of 81 participants (see **Figure 1**). To increase diagnostic specificity, we excluded individuals with suspected vascular, alcohol-related dementia or other dementia subtypes based on detailed clinical history and cognitive profiles.

For the calculation of the sample size, we used the Aβ42/40 levels from the laboratory of Dr. Teunissen (co-investigator), who found in our preliminary study (35), a mean of 0.06 (standard deviation = 0.02) in 241 amyloid-negative controls and a mean of 0.05 (standard deviation = 0.01) in 297 patients with Alzheimer’s-type dementia (effect size = 0.01). Assuming a pooled standard deviation of 0.02 units, the study would require a sample of 63 subjects per group (i.e., a total sample of 126 subjects, assuming groups of equal size) to obtain a power of 80% and a significance level of 5% (two-sided), in order to detect a true difference of 0.01 units between the means of the suspected dementia group and the control group. Therefore, with a sample of 81 participants with dementia (75 participants in a group is sufficient), we will have adequate power to determine significant differences in biomarker values between cognitively normal controls and individuals with suspected dementia. This sample is good for the present study and allows conclusions to be drawn.

### 2.4 Measures

#### 2.4.1 Community-Based Dementia Screening Instrument (CSID)

Although not a hard and fast reference, the CSID is widely used in developing countries where validated clinical diagnostic tools are limited (38). It has demonstrated strong utility in many international studies of dementia in sub-Saharan Africa (39–42). The CSID includes an informant section and a cognitive assessment section. The cognitive part assesses orientation, attention, language, constructive practice, learning, and memory (via 10-word learning trials and delayed recall). Scores range from 0 to 55, with lower scores indicating lower cognitive performance. The instrument offers excellent inter-rater reliability (0.99).

#### 2.4.2 Alzheimer’s Questionnaire (AQ)

AQ assesses orientation, motor function, language, visuospatial abilities, and memory. This yes/no questionnaire gives a score from 0 to 26, with higher scores indicating greater disability (44, 46). Its diagnostic performance is robust, with a reported sensitivity of 98.55% and a specificity of 96% to identify AD (44). The choice to use only the total AQ score is based on the fact that it is a validated overall indicator of the severity of the disorders assessed.

#### 2.4.3 Geriatric Depression Scale

The 15-item GDS is a widely used self-report tool for depression in older adults (47). Total scores range from 0 to 15, with higher scores reflecting more severe depressive symptoms. A score ≥5 is commonly used as a cut-off for clinically significant depression (48, 49). GDS demonstrates high diagnostic accuracy, with a sensitivity of 92% and a specificity of 89% (50).

#### 2.4.4 Plasma biomarkers

Blood samples were collected at the Kinshasa Medical Center (CMK) blood transfusion laboratory by antecubital venipuncture in EDTA dipotassium tubes (K_2_EDTA). Samples were centrifuged at 1800 g for 15 minutes at room temperature, and 5 mL of plasma was aliquoted in 0.5 mL polypropylene tubes. Plasma was initially stored at −20 °C for less than a week, then transferred to a freezer at −80 °C for long-term storage (51). The aliquots were shipped frozen on dry ice to Emory University for storage, and then to the University of California, San Francisco (UCSF) for biomarker analysis.

Biomarkers were measured using the commercially available Quanterix kits: Neurology 4-PLEX E (Aβ40, Aβ42, Nfl and GFAP; lot 503819), P-Tau181 (P-Tau181 v2; lot 503732), IL-1β (lot 503806) and IL-10 (IL-10 2.0; lot 503533), on the Simoa HD-X platform (Billerica, MA) at UCSF. P-tau217 was measured using the proprietary ALZpath pTau-217 CARe Advantage kit (Lot No. MAB231122, ALZpath, Inc.) on the Simoa HD-X platform (68). All analytes were measured in duplicate, with the exception of IL-1β, which was measured in single due to the small sample quantity (52, 53). For Aβ40, Aβ42, Nfl and GFAP, all samples had concentrations above the lower limit of quantification (LIQ) of 1.02 pg/mL, 0.378 pg/mL, 0.4 pg/mL and 2.89 pg/mL, respectively. The average coefficients of variation (CVs) were 6.0%, 6.5%, 5% and 4.6%, respectively. For P-Tau181, all samples had concentrations above the kit’s lower limit of quantification (LOQ) of 0.085 pg/mL, with an average CV of 11.6%. The IQs for IL-1β and IL-10 were 0.083 pg/mL and 0.021 pg/mL, respectively. The mean coefficient of variation for IL-10 was 6.1% (54, 55). The preliminary cut-off values for plasma concentrations (in pg/mL) were: 0.061 for Aβ42/40, 4.50 for p-tau181, 0.008 for Nfl, 176 for GFAP, 1.16 for TNFα, 0.011 for IL-1β and 0.38 for IL-10. All AUCs ranged from 0.64 to 0.74 (54).

### 2.5 Ethical considerations

All ethical and confidentiality standards have been respected. The study received prior approval from the ethics committee of the School of Public Health of the University of Kinshasa. All participants have given written informed consent prior to registration.

### 2.6 Statistical analysis

Statistical analyses were performed with R version 4.4.0. Descriptive statistics (means and standard deviations) were used for continuous variables, and frequencies or percentages for categorical variables. For interpretability, the continuous GDS score was dichotomized, with the GDS ≥ 5 indicating clinically significant depression (48).

Cognitive test scores were analyzed using multiple linear regression models adjusted for sex, age, and education level. To examine whether plasma biomarkers predicted the presence of depression, logistic regression models were used, with biomarkers as predictors and depressive state as the dependent variable. Odds ratios (ORs), 95% confidence intervals (CIs) and p-values were reported, with statistical significance set at p < 0.05.

## 3 Results

The demographic, clinical, and baseline characteristics of the plasma biomarkers, stratified by the neurological status of the sample, are presented in **Table 1**. The mean age of the population was 72.9 years with a standard deviation of 7.7 years. The mean age in the group of people suspected of AD was 73.8 years with a standard deviation of 7.55 years. Regarding gender, women represented 54.6% of the sample and 55.6% had clinical dementia. The education level of this population was 8.45 years and older in cognitively healthy individuals aged 9.58 years. In addition, 80% of the sample had symptoms of geriatric depression in the group with suspected AD, compared to 22% in the cognitively healthy (HC) control group. The Aβ42/40 ratio in the sample was lowest at 0.069, and the concentrations of GFAP, Aβ40 and NfL in the sample were highest at 205.9 pg/mL, 72.9 pg/mL and 50.6 pg/mL, respectively.

**Table 1:** Demographic characteristics, medical, clinical, neuropsychological history, and biomarkers of the sample stratified by neurological status.

	Clinical dementia (n = 45)	Healthy controls (n=41)	Total (n=86)	
Data demographic				
Age	73.8 (7.55)	71.9 (7.78)	72.9 (7.7)	
Female, n (%)	25 (55.6%)	22 (53.7%)	47 (54.6%)	
Education	7.44 (5.51)	9.58 (5.15)	8.45 (5.42)	
Depression/depressive symptoms				
GDS	7.47 (3.45)	3.67 (2.32)	5.70 (3.53)	
≥5	36 (80.0%)	9 (22.0%)	45 (52.3%)	
<5	9 (20.0%)	30 (73.2%)	39 (45.4%)	
Plasma biomarkers				
Aβ_40_ (pg/ml)	78.4 (50.1)	3.83 (2.31)	3.82 (2.14)	
Aβ_42_ (pg/ml)	3.81 (2.02)	68.1 (51.6)	72.9 (50.7)	
Aβ_42/40_	0.062 (0.030)	0.078 (0.040)	0.069 (0.036)	
p-tau181 (pg/ml)	3.03 (2.26)	2.24 (1.50)	2.67 (1.97)	
Aβ_40_/ p-tau181	36.8 (47.4)	70.6 (184.6)	52.7 (131.4)	
NfL (pg/ml)	62.7 (41.5)	37.3 (31.4)	50.6 (39.0)	
GFAP (pg/ml)	241.0 (143.6)	167.3 (98.3)	205.9 (128.8)	
TNF-α (pg/ml)	0.58 (0.23)	0.62 (0.33)	0.60 (0.28)	
IL-10 (pg/ml)	0.27 (0.34)	0.35 (0.36)	0.31 (0.35)	
IL-1β (pg/ml)	0.27 (0.34)	0.35 (0.36)	0.31 (0.35)	

Variable, n (%)	Clinical dementia (n = 43)	Healthy controls (n = 38)	Total (n = 81)	
Medical history				
High blood pressure	26 (60%)	17 (45%)	43 (53%)	
High cholesterol	1 (2%)	1 (3%)	2 (2%)	
Poor diet	1 (2%)	0 (0%)	1 (1%)	
Stroke	2 (5%)	0 (0%)	2 (2%)	
Tobacco abuse	4 (9%)	5 (13%)	9 (11%)	
Alcohol abuse	12 (28%)	4 (11%)	16 (20%)	
Anxiety	3 (7%)	2 (5%)	5 (6%)	

Variables μ (σ)	Clinical dementia (n= 45)	Healthy controls (N=39)	Total (n=84)	P-value
Screening and cognitive tests				
CSID	19.6 (5.6)	31.9 (2.8)	24,9 (7,6)	<.0001
AQ	19.3 (4.0)	3.2 (2.7)	12,1 (8,8)	<.0001
ANT	15.5 (7.2)	21.7 (4.1)	18,5 (6,7)	<.0001
ALMT Trial 1	2.6 (1.7)	5.5 (1.6)	4,0 (2,2)	<.0001
ALMT Test 3	4.0 (1.9)	7.8 (2.0)	5,8 (2,7)	<.0001
ALMT reminder				
AVMT Trial 1	0.31 (0.64)	6.7 (1.6)	3,3 (3,4)	<.0001
AVMT Trial 3	1.3 (1.6)	4.2 (2.9)	2,7 (2,7)	<.0001
AVMT Callback	1.9 (1.9)	8.5 (4.5)	5,0 (4,7)	<.0001
Proverb Test	1.0 (1.7)	7.8 (4.3)	4,3 (4,7)	<.0001
African Card Game	2.5 (2.2)	6.9 (5.1)	4,6 (4,4)	<.0001
Winnings	21.7 (7.0)	28.2 (9.8)	24,8 (9,0)	0.021

Values in parentheses indicate the standard deviation. Statistical analyses were performed using linear regression adjusted for age, sex, and education.

Aβ, amyloids-β ; GDS, Geriatric Depression Scale; GFAP, Glial Fibrillar Acid Protein; IL-1β, Interleukin-1β, IL-10, Interleukin-10; NfL, neurofilament light chain; p-tau: phosphorylated tau; TNF-α, tumor necrosis factor-α.

ALMT, African List Memory Test; ANT, African Naming Test; AQ, Alzheimer questionnaire, AVMT, African Visuospatial Memory Test; CSID, a community-based dementia screening tool.

Regarding the medical history of the sample population, hypertension (hypertension) was very common in individuals suspected of AD at 60%, followed by alcohol abuse, present in 28% of the group of individuals suspected of AD compared to 11% in the cognitively healthy control group, and tobacco abuse at 9% in the group suspected of AD, compared to 13% in the cognitively healthy control group.

Cognitively healthy individuals scored significantly higher than those with clinical dementia, controlling for age, gender, and education level.

**Table 2** presents the plasma biomarkers associated with the occurrence of symptoms of depression or geriatric depression. In this study, among all plasma biomarkers analyzed, only nonspecific biomarkers (NfL and GFAP) showed a significant association with depression in older adults with probable AD. Higher concentrations of NfL (OR = 1.76; p = 0.04) and GFAP (OR = 1.98; p = 0.02) show an association with an increased likelihood of geriatric depression.

**Table 2:** Prediction of depression using plasma biomarkers in older adults with probable alzheimer’s disease.

Plasma biomarkers	Depression	
	OR (95% CI)	P-value
Aβ42/40	0.76 (0.46-1.23)	0.27
p-tau 181	1.26 (0.78-2.08)	0.34
NfL	1.76 (1.06-3.20)	0.04
GFAP	1.98 (1.17- 3.67)	0.02
IL-1β	0.77 (0.47-1.20)	0.25
α TNF-	0.83 (0.52-1.31)	0.43

## 4 Discussion

This study examined the association between plasma neurodegenerative biomarkers and the occurrence of depression in older adults with or without Alzheimer’s dementia in Kinshasa, Democratic Republic of Congo (DRC). To our knowledge, this is one of the first exploratory investigations in sub-Saharan Africa (SSA) — and more specifically in the DRC — to assess whether plasma biomarkers of AD are associated with geriatric depression. We hypothesized that disease-specific biomarkers of AD. (Aβ and p-Tau) are associated with depression, regardless of dementia status.

Our results indicate a higher prevalence of depression in people with dementia compared to healthy controls. However, only nonspecific biomarkers—neurofilament light chain (NfL) and glial fibrillar acid protein (GFAP)—were significantly associated with geriatric depression. This demonstrates that an association was observed between high concentrations of NfL and GFAP on the one hand and an increased likelihood of depression on the other, while AD-specific biomarkers did not show significant associations. Thus, our initial hypothesis was only partially substantiated.

The high prevalence of depressive symptoms in people with dementia is consistent with previous studies. Guerchet et al. (2012) and other researchers reported a strong association between depressive symptoms and suspicion of dementia in SSA populations (1–4), and Steinberg et al. (2008) found that up to two-thirds of people with dementia develop depression (56). This high prevalence may reflect psychological reactions to cognitive decline, loss of autonomy and functional impairment (57, 58). Biological mechanisms—including neuronal degeneration in mood-regulating regions, neuroinflammation, and decreased social support—may further worsen depressive symptoms in AD (59, 60).

Regarding biomarkers, our findings are consistent with recent findings by Schuurmans et al. (2024), who reported that higher levels of NfL were associated with more depressive symptoms and an increased risk of incident depressive episodes, including major depressive disorder (61, 62). Similarly, Guo and Zhu (2024) found that high levels of serum NfL predicted depressive symptoms (63). These findings support the hypothesis that neuroaxonal damage contributes to late-onset depression.

Although few studies have examined GFAP in relation to depression, the existing evidence supports our observations. GFAP has been associated with neuropsychiatric symptoms, including depression, and may serve as a marker of astroglial activation and neuroinflammation (23, 24). Elevated levels of GFAP have been associated with increased age, greater depressive severity, and inflammatory processes involved in mood disorders (24, 28). Bacci et al. (2026), also demonstrated that GFAP was associated with depressive symptoms. Neuroinflammation may explain the depressive symptoms in this group (64), its involvement in neurodegeneration and depression, often associated in the elderly, has also been well demonstrated (65, 66). In addition, GFAP may also help differentiate major depressive disorder (MDD) and monitor astroglial pathology (24).

The association between NfL, GFAP and depression in our sample could also reflect the high vascular burden in the DRC. Hypertension was widespread (60% in the Alzheimer’s group), and both biomarkers are sensitive to small vessel disease and neurodegeneration—key factors in end-of-life vascular depression.

In contrast, most North American studies have focused on AD-specific biomarkers (13, 15, 67). Harrington et al. (2017) reported that elevated Aβ levels increased the likelihood of clinically significant depressive symptoms (11), and Yasuno et al. (2016) found a positive correlation between amyloid plaque load and depression (16). Other studies have highlighted tau pathology as a stronger correlate of depression than amyloid (13, 19, 20). These discrepancies may stem from methodological differences: many Western studies use PET imaging or CSF biomarkers, which more directly reflect the central pathology, while plasma biomarkers may have a lower sensitivity to detecting brain changes. In addition, most of the studies cited are longitudinal, while ours is cross-sectional.

## 5 Limitations and future directions

This study has several limitations. First, the sample size was modest, due in part to cultural hesitancy towards blood sampling and the mistrust of potential participants. This can limit statistical power and reduce generalizability. Second, we included only cognitively normal individuals and those with dementia, excluding intermediate cognitive states such as MCI or subjective cognitive impairment. This decision was driven by budgetary constraints and case-control design but limits our ability to examine biomarker trajectories across the cognitive continuum.

Third, the screening tools used (CSID, QA, and GDS), although widely applied in AHS research, have not been formally validated in the region. This may have led to misclassification of dementia subtypes or depressive symptoms. Finally, plasma biomarkers may not fully capture central neuropathological processes, especially compared to CSF or PET-based measurements.

Future studies should include larger and more diverse samples and incorporate all cognitive stages (MCI, subjective cognitive complaints, AD, and other dementias). The validation of cognitive and depressive screening tools for SSA populations is also essential. Longitudinal designs and multimodal biomarker approaches would further clarify the causal pathways between neurodegeneration and depression.

Finally, certain potential confounding factors (hypertension, vascular risk factors, alcohol consumption) were not included in the regression models due to limitations related to the quality and reliability of the collected data, which were primarily based on participants’ self-reports in a sociocultural context where information is often reported in a non-standardized manner. Data on anti-inflammatory drug use prior to blood collection were not collected in this study. Future studies should account for potential confounding factors, as anti-inflammatory drug use could influence certain biomarkers, particularly cytokines.

## 6 Highlights of the study

Despite its limitations, this study provides one of the first datasets linking plasma biomarkers and depression in older adults in SSA. Our findings highlight that nonspecific biomarkers — rather than AD-specific markers — are associated with depression in people suspected of having AD. These findings provide a basis for future research on biomarkers in African contexts and highlight the importance of integrating biomarker assessments into diagnostic and prognostic assessments in older adults with mood and cognitive disorders.

In addition, the study highlights the need to integrate geriatric depression into national dementia strategies and to support research on plasma biomarkers to facilitate their future integration into clinical practice in the DRC and across SSA.

## Data Availability

The raw data supporting the conclusions of this article will be made available by the authors, without undue reservation.
